# Juvenile and adult open-angle glaucoma form a single polygenic continuum

**DOI:** 10.64898/2026.09.13.26361381

**Published:** 2026-09-14

**Authors:** Joshua Schmidt, Ma. Carmela Guevarra, Guiyan Ni, Thomas A. D. Hassall, Shannon Ji, Thi T. Nguyen, Georgina L. Hollitt, Antonia Kolovos, Emmanuelle Souzeau, Ngoc Quynh Le, Puya Gharahkhani, Jason Turner-Maier, Edward Ryan A. Collantes, Inas F. Aboobakar, Ayellet V. Segrè, Alex W. Hewitt, Nick Haan, Stuart MacGregor, Janey L. Wiggs, Jamie E. Craig, Owen M. Siggs

## Abstract

**Purpose:**

To test whether juvenile open-angle glaucoma (JOAG) and primary congenital glaucoma (PCG) are polygenically continuous with primary open-angle glaucoma (POAG), using a multi-trait POAG polygenic risk score (PRS).

**Design:**

Retrospective cohort study.

**Participants:**

3,102 open-angle glaucoma index cases, of whom 2,836 had a recorded age at diagnosis (52 PCG, 226 JOAG, and 2,558 POAG) and 999 European-ancestry controls from a glaucoma disease registry and a population-based cohort. Two replication cohorts comprised 70 JOAG and 31 PCG probands with 230 controls (MEE), and 9 PCG and 120 POAG cases (GOGS).

**Methods:**

One index case per family was analysed. PRS were calculated from weighted SNPs and adjusted for ancestry. Cases were stratified by age at diagnosis (PCG ≤3 years, JOAG ≥4 and <40 years, POAG ≥40 years) and presence of pathogenic/likely pathogenic variants (“Mendelian”). Groups were compared by Kruskal-Wallis test, with logistic regression and area under the curve for per-SD effects.

**Main Outcome Measures:**

PRS centile values compared across glaucoma subtypes and Mendelian and non-Mendelian subgroups.

**Results:**

Mean PRS was highest in cases diagnosed in the fourth decade of life. Among subgroups, it was highest in non-Mendelian JOAG (84.1%), exceeding controls (53.3%), Mendelian JOAG (68.9%), and non-Mendelian POAG (79.3%) (all P ≤ 0.003). Non-Mendelian JOAG was also the best discriminated from controls (OR 3.66 per SD increase, 95% CI 3.02-4.49; AUC 0.814, 95% CI 0.782-0.845). Neither PCG subgroup differed from controls. Among Mendelian cases, only *MYOC* p.Gln368Ter showed significantly elevated PRS (OR 2.49 per SD, 95% CI 1.89-3.34). In the MEE replication cohort, JOAG was associated with higher PRS (OR 2.12 per SD, 95% CI 1.50-3.07) while PCG was not (OR 0.90, P = 0.73). In GOGS, mean PRS was higher in POAG than PCG (84.8 vs 59.7 centile; P = 0.026).

**Conclusions:**

A POAG PRS predicts JOAG risk with discrimination comparable to or greater than POAG itself, supporting a shared polygenic architecture across juvenile and adult open-angle glaucoma. PCG showed no PRS elevation regardless of Mendelian status, indicating a distinct architecture for which rare-variant sequencing remains appropriate.

## Full Text Availability

The license terms selected by the author(s) for this preprint version do not permit archiving in PMC. The full text is available from the preprint server.

